# International EANM-SNMMI-ISMRM consensus recommendation for PET/MRI in oncology

**DOI:** 10.1007/s00259-023-06406-x

**Published:** 2023-08-25

**Authors:** Patrick Veit-Haibach, Håkan Ahlström, Ronald Boellaard, Roberto C. Delgado Bolton, Swen Hesse, Thomas Hope, Martin W. Huellner, Andrei Iagaru, Geoffrey B. Johnson, Andreas Kjaer, Ian Law, Ur Metser, Harald H. Quick, Bernhard Sattler, Lale Umutlu, Greg Zaharchuk, Ken Herrmann

**Affiliations:** 1 Joint Department Medical Imaging, University Health Network, Mount Sinai Hospital and Women’s College Hospital, Toronto General Hospital, 1 PMB-275, 585 University Avenue, Toronto, Ontario M5G 2N2 Canada; 2 Joint Department of Medical Imaging, University of Toronto, Toronto, Canada; 3 Department of Surgical Sciences, Uppsala University, 751 85 Uppsala, Sweden; 4 Antaros Medical AB, BioVenture Hub, 431 53 Mölndal, Sweden; 5 Department of Nuclear Medicine and Molecular Imaging, University Medical Center Groningen, Groningen, The Netherlands; 6 Department of Radiology and Nuclear Medicine, VU University Medical Center, Amsterdam, The Netherlands; 7 Department of Diagnostic Imaging (Radiology) and Nuclear Medicine, University Hospital San Pedro and Centre for Biomedical Research of La Rioja (CIBIR), Logroño, La Rioja Spain; 8 Department of Nuclear Medicine, University of Leipzig Medical Center, Leipzig, Germany; 9 Department of Radiology and Biomedical Imaging, University of California San Francisco, San Francisco, CA USA; 10 Department of Nuclear Medicine, University Hospital Zürich, University of Zürich, Rämistrasse 100, 8091 Zurich, Switzerland; 11 Department of Radiology, Division of Nuclear Medicine, Stanford University Medical Center, Stanford, CA USA; 12 Division of Nuclear Medicine, Department of Radiology, Mayo Clinic, Rochester, MN USA; 13 Department of Clinical Physiology, Nuclear Medicine & PET and Cluster for Molecular Imaging, Rigshospitalet and University of Copenhagen, Copenhagen, Denmark; 14 Department of Clinical Physiology, Nuclear Medicine & PET, Rigshospitalet, Copenhagen, Denmark; 15 Joint Department of Medical Imaging, University Health Network, Mount Sinai Hospital and Women’s College Hospital, University of Toronto, Toronto, Ontario Canada; 16 High-Field and Hybrid MR Imaging, University Hospital Essen, University of Duisburg-Essen, Essen, Germany; 17 Erwin L. Hahn Institute for MR Imaging, University of Duisburg-Essen, Essen, Germany; 18 Department of Nuclear Medicine, University Hospital Leipzig, Leipzig, Germany; 19 Department of Diagnostic and Interventional Radiology and Neuroradiology, University Hospital Essen, Essen, Germany; 20 Division of Neuroradiology, Department of Radiology, Stanford University, 300 Pasteur Drive, Room S047, Stanford, CA 94305-5105 USA; 21 Department of Nuclear Medicine, University of Duisburg-Essen and German Cancer Consortium (DKTK), University Hospital Essen, Essen, Germany

**Keywords:** Consensus, Oncology, SNMMI, ISMRM, EANM, PETMR, PET, MRI

## Abstract

The Society of Nuclear Medicine and Molecular Imaging (SNMMI) is an international scientific and professional organization founded in 1954 to promote the science, technology, and practical application of nuclear medicine. The European Association of Nuclear Medicine (EANM) is a professional non-profit medical association that facilitates communication worldwide between individuals pursuing clinical and research excellence in nuclear medicine. The EANM was founded in 1985. The merged International Society for Magnetic Resonance in Medicine (ISMRM) is an international, nonprofit, scientific association whose purpose is to promote communication, research, development, and applications in the field of magnetic resonance in medicine and biology and other related topics and to develop and provide channels and facilities for continuing education in the field.The ISMRM was founded in 1994 through the merger of the Society of Magnetic Resonance in Medicine and the Society of Magnetic Resonance Imaging. SNMMI, ISMRM, and EANM members are physicians, technologists, and scientists specializing in the research and practice of nuclear medicine and/or magnetic resonance imaging.

The SNMMI, ISMRM, and EANM will periodically define new guidelines for nuclear medicine practice to help advance the science of nuclear medicine and/or magnetic resonance imaging and to improve the quality of service to patients throughout the world. Existing practice guidelines will be reviewed for revision or renewal, as appropriate, on their fifth anniversary or sooner, if indicated. Each practice guideline, representing a policy statement by the SNMMI/EANM/ISMRM, has undergone a thorough consensus process in which it has been subjected to extensive review. The SNMMI, ISMRM, and EANM recognize that the safe and effective use of diagnostic nuclear medicine imaging and magnetic resonance imaging requires specific training, skills, and techniques, as described in each document. Reproduction or modification of the published practice guideline by those entities not providing these services is not authorized.

These guidelines are an educational tool designed to assist practitioners in providing appropriate care for patients. They are not inflexible rules or requirements of practice and are not intended, nor should they be used, to establish a legal standard of care. For these reasons and those set forth below, the SNMMI, the ISMRM, and the EANM caution against the use of these guidelines in litigation in which the clinical decisions of a practitioner are called into question.

The ultimate judgment regarding the propriety of any specific procedure or course of action must be made by the physician or medical physicist in light of all the circumstances presented. Thus, there is no implication that an approach differing from the guidelines, standing alone, is below the standard of care. To the contrary, a conscientious practitioner may responsibly adopt a course of action different from that set forth in the guidelines when, in the reasonable judgment of the practitioner, such course of action is indicated by the condition of the patient, limitations of available resources, or advances in knowledge or technology subsequent to publication of the guidelines.

The practice of medicine includes both the art and the science of the prevention, diagnosis, alleviation, and treatment of disease. The variety and complexity of human conditions make it impossible to always reach the most appropriate diagnosis or to predict with certainty a particular response to treatment.

Therefore, it should be recognized that adherence to these guidelines will not ensure an accurate diagnosis or a successful outcome. All that should be expected is that the practitioner will follow a reasonable course of action based on current knowledge, available resources, and the needs of the patient to deliver effective and safe medical care. The sole purpose of these guidelines is to assist practitioners in achieving this objective.

## Introduction

Positron emission tomography/magnetic resonance imaging (PET/MRI) is the latest combination of noninvasive diagnostic positron emission tomography (PET) imaging. As other PET-based imaging procedures, it provides tomographic images of metabolic activity or other phenotypic characteristics of target tissues and offers the option of (semi)quantification. While most guidelines on PET imaging are radiopharmaceutical-focused (i.e., [^18^F]fluorodeoxyglucose-PET/CT imaging, [^68^Ga]Ga-DOTA-based PET-imaging), this PET/MRI guideline is technology oriented. Also, the radiopharmaceutical and PET related part is mostly not significantly different from PET/CT and other radiopharmaceutical-based guidelines. Therefore, we will always refer for those parts to the respective PET/CT guideline. Thus, in opposition to other guidelines, the authors more emphasize on the intersection of MRI and PET imaging, the combined PET/MRI acquisition and its clinical uses. The authors would also like to point out that this work is constantly evolving, that this is not a definitive document, and this document might well serve as the offspring for other consecutive, disease-based and tracer-based and more individualized PET/MRI guidelines.

As for the approval process, this document went through the standardized combined approval procedure of the European Association of Nuclear Medicine (EANM) and the Society of Nuclear Medicine (SNM). This includes submission to the respective committees within the EANM and SNM. After review and revision based on the committees’ comments, the documents underwent review by the national delegates of the EANM. After iterative revision, the document was submitted to the PET MR Study Group of the International Society for Magnetic Resonance in Medicine (ISMRM) for approval. After that, it went through the Publications Committee and the Board of Trustees of the ISMRM before final submission. The authors from all mentioned societies participated in drafting and revision of the manuscript.

## Goals

Currently, there are multiple studies available on the evaluation of different diseases with PET/MR. However, there is an information gap on how to actually perform the PET/MR itself in a somewhat harmonized way. The objective is to share a basic technical acquisition groundwork and to provide a minimum agreeable standard imaging protocol for the main PET/MRI indications in oncology. This guideline will give not any fixed recommendations; instead, it will suggest where PET/MRI should be used instead of PET/CT or where PET/MRI should be used instead of MRI + PET/CT since there are no clear recommendations for such scenarios available in the literature. The purpose of this guideline is also to provide PET/MRI users with a common denominator for recommending, performing, interpreting, and reporting the results of the most used PET/MRI indications. It is not intended to prescribe users with fixed and inflexible protocols. It is designed and meant to rather describe the technological possibilities and how to properly adapt the combined acquisition techniques according to clinical indications.

As in PET/CT, the user is able to use the PET component in PET/MRI as a (semi)quantitative imaging technique. In addition, a variety of partly also quantitative MRI techniques can be acquired simultaneously. Thus, PET/MRI also requires standardized quality control (QC)/quality assurance (QA) procedures to maintain the accuracy and precision of quantitation for both procedures [1, 2]. These quality measures are essential preconditions to ensure repeatability and reproducibility. Reliable repeatability and reproducibility are necessary requirements for the clinical management of patients but also for comparability in multicenter trials. Common and standardized combined imaging protocols are important to promote the appropriate use of this complex combined imaging procedure but also to increase the awareness about the advantages of this first truly simultaneous hybrid, cross-modality molecular imaging method. It also ensures accurate evaluation of disease staging, response, and follow-up. This guideline has no direct predecessor, however, especially the PET part is partly building on previously published guidelines, i.e., PET/CT for tumor imaging and the SNMMI procedure guidelines for tumor imaging 1.0 [1, 3, 4].

## PET/MRI technology fundamentals and quality controls

Positron emission tomography and magnetic resonance imaging (PET and MRI) have been each in the arena of clinical molecular tomographic imaging more than three decades. While MRI was originally aimed at imaging soft tissue structures and PET was primarily aimed at imaging physiologic and pathophysiologic processes using Fluorodeoxyglucose (FDG), both gradually expanded. MRI gained molecular imaging capabilities beyond morphology and PET increased its offer of radiopharmaceuticals to image oncologic and non-oncologic targets.

After the proof of operability of specific light detectors such as avalanche photo diodes (APD) and silicon photo multipliers (SiPM) in a magnetic field, the way of combining PET and MRI to hybrid systems was paved. First, PET-detector inserts for preclinical [5–11] and clinical MR–systems [12, 13] were developed to further proof the feasibility of combining two imaging modalities without impairing the qualitative and quantitative imaging results intolerably. In parallel, by using systems employing a single patient handling system/table for either imaging modality, the clinical utility of having the image data co-registered was thoroughly investigated. Regarding the latter, GE Healthcare devised a two-room-setup where a clinical PET/CT and an MRI system were installed in rooms right next to each other and the patient handling system designed connectable to the patient ports/-bores of both of the systems [14, 15]. One step further, Philips omitted the CT component and introduced a bi-planar system installing the PET- and the MRI-gantry in one room using a rotating patient table [16, 17]. Finally, the first system fully integrating the APD based PET detector system was designed by Siemens Healthineers and reached market maturity in 2010 [18–20]. This system offered the option of true simultaneous whole-body measurement of PET and MRI signals. However, the limited timing resolution of the APDs did not allow for time of flight (TOF) PET measurements as utilized in photomultiplier-based PET detector systems in state-of-the-art hybrid PET/CT systems. In 2013, GE healthcare introduced a TOF-PET-capable fully integrated whole-body PET/MRI system employing SiPMs as light detectors in the PET-detector system [21]. In a comprehensive overview, Quick and Boellaard summarized the performance parameters of the three PET/MRI systems available for clinical use [16, 22]. SiPM based detectors are also used in digital PET detector systems that are employed in hybrid PET/CT to further increase timing resolution and reduce the influence of noise by analog–digital conversion. “Digital” in this context means that each light photon is detected by the SiPM and directly converted into a binary output signal (i.e., output = 1 → photon detected, output = 0 → no photon detected). As a consequence, a considerable number of SiPM-microcells read out the scintillation light of one particular detector crystal. Each individual crystal can be read out eliminating the need for localization electronics and arrays within the detector block electronics. Since there is no analog–digital conversion involved in this process and each light photon leads to a binary output of a SiPM cell, this method can be called digital photon counting (DPC). More technical details and performance characteristics can be found in the article by Seifert et al. as well as in the referenced book [23].

Depending on the legislation and organizational requirements and settings, PET/MRI systems should usually be installed in and managed by nuclear medicine, radiology, or both departments altogether. If it is integrated in a full hybrid imaging molecular imaging PET center with a radiopharmaceutical production site including a cyclotron, the system should be located in close proximity to this facility. However, satellite PET/MRI units can also be installed at specialized clinical sites such as pediatric, psychiatric, or neurology departments if the proper staffing level is available.

## Qualifications and responsibilities of personnel

When PET/MRI as a cross-modality hybrid imaging system is put into operation, the availability of adequately trained personnel must also be planned in advance. This obviously refers to both the PET and the MRI components of the integrated system and to all professional groups involved (physicians, physicists, technologists) [19]. Technologists in charge of running the daily operation should be well trained and experienced in operating both modalities. Depending on the local workload and the level of training and experience, two individuals might be necessary to operate the system effectively. Curricula of technologist education are country dependent. While some countries may include training in understanding and the operation of all radiological (including MRI), radiotherapy, and nuclear medicine (NM) imaging as well as non-imaging equipment (academic or non-academic), other countries may only focus their training on the modality the technologist is obtaining licensure in. However, the level of actual practical experience will depend on the main site of deployment in one of the aforementioned fields. So, for instance, technologists working mainly in the MRI field might not be fully aware of the issues with handling radioactivity, the respective molecular imaging equipment and radiation protection, and, vice versa, technologists coming from the nuclear medicine molecular imaging and therapy field might not be fully aware of the issues and safety precautions with high-field magnetism, radiofrequency transmission as well as understanding, and adapting MRI sequences and protocols.

In principle, all this is also true for physicians and other academic personnel involved. In general, to justify, conduct, analyze, and report MRI and PET, physicians specialized in those particular fields need to be involved. Generally, this is achieved by completing the curricula in Radiology and Nuclear Medicine resulting in specialization as a radiologist, a nuclear medicine physician or both. The integration of these specializations varies considerably from a full integration into one curriculum to two separate specializations from country to country. Once a hybrid oncology PET/MRI has been successfully acquired and analyzed, a joined reading and reporting should be conducted. Ideally, this is done in regular conferences of the MRI and PET-specialized physicians where results including a joined conclusion are finalized in one report. Additionally, the complexity of the methodology and the level of sophistication requires the availability of physicists and/or engineers experienced and specialized in one or both fields from a physical-technical perspective.

Another aspect is that in most of the countries fulfillment of regulatory requirements, particularly when handling radioactive material in clinical and/or research settings, is mandatory. This includes specialization and licensing of the involved non-academic (technologists, nurses) and academic (physicians, physicists, engineers, technologists) personnel including documented regular knowledge refreshment and continuing education.

### Quality assurance and control of PET (QA/QC)

Generally, the quality control of the PET system should follow the specifications of the manufacturer. The system should be turned off and rebooted daily to allow for a full reset of all hardware and software components. The daily reboot comprises a basic check of important parameters of components, internal and external connections between those as well as all precautions relevant to the operators and patients’ safety. Right after the reboot, calibration check and normalization of the PET detector system must follow. This is usually carried out using a Gallium-68 filled cylindrical phantom that is placed in the center of the PET FOV. This quality check should follow a protocol predefined by the manufacturer and result in an output of a sinogram for visual inspection accompanied by information to the user to check if sensitivity and calibration of the PET detector system is within acceptable levels. The results and the trend of this test over time are stored internally and the user is only alerted if the results are outside of certain specifications. It is advisable to the user to read this trend periodically and keep it for their own records. Usually, the calibration and sensitivity are very stable and need to be recalibrated only on occasion of planned maintenance by the manufacturer or on occasion of major repair service events including replacement of detector modules or electronics for instance. Less frequent necessary QA/QC procedures can be found in the EANM recommendations on routine quality control for nuclear medicine instrumentation [24], an IAEA publication on the topic [25], the NEMA NU2 2012 publication [26], or international and national standards. In most countries, the basic QA/QC measures of PET systems are written in legislation and mandatory before the system can be used for patient care. Sattler et al. extracted the most important QA/QC measures as applicable for combined/simultaneous PET/MRI systems [19]. The accuracy of PET quantification decisively depends on the correct system calibration involving all corrections that are implicitly necessary using the full ring PET detector setting in 3D mode. Basically, these are scatter, random, attenuation, and dead-time corrections and detector normalization on a regular basis. They are required for correct activity quantification in a lesion, organ, or phantom. Ultimately, these measures are influenced by correct attenuation correction (AC). Dependent on the body region under investigation, there are several methods to generate a map of linear attenuation coefficients (LAC) [27, 28]. As an important QC measure, these maps are to be at least visually inspected for every investigation before reconstruction. Finally, analysis and quantification of PET data can be approved.

### Quality control of MRI prior to scanning

In contrast to the requirements for the PET component of the combined system, there is no legal requirement to perform standardized QC/QA measures of the MRI part. However, some regular (not daily as in PET-imaging) basic performance tests are advisable to ensure appropriate imaging performance and patient safety. A comprehensive set of tests is described in the MRI Accreditation Program Requirements of the ACR [29]. Regarding the strong magnetic and electromagnetic fields involved in MRI, patient safety procedures need to be established in the entire PET/MRI setting and in the routine patient workflow. Here, we refer to the section on patient preparation and MRI safety below.

## Procedure/specifications of the examination

### Request/referral/justifying indications

Patient referral for PET/MRI is determined by the interplay and communication between the referring oncologic centers, institutions, and physicians. Due to the complexity of the method, especially on the PET part, referring physicians should communicate with the PET/MRI responsible physician(s) well in advance. Be it on the occasion of interdisciplinary tumor boards or through other dedicated means of communication, agreement with regard to the eligibility of the particular patient should be reached based on previous clinical findings in order to justify the investigation and optimize the exam protocol. Thus, it is ensured an optimal comprehensive answer to the clinical question by the PET/MRI investigation. If patients need to be sedated or anesthetized for the imaging procedure, particular scrutiny is needed to optimally adapt the schedule of both procedures (anesthesia and imaging). All intensive care equipment that has to be brought in the PET/MRI suite must be certified as MRI compatible. The request or referral for a PET/MRI should be directed to the department which is responsible for the PET/MRI operations. As in PET/CT, it should contain appropriate medical information to justify the medical need for the examination, including diagnosis, the specific medical question, and a brief medical history. Especially the specific medical question is of utmost importance in PET/MRI since it defines the acquisition protocol. In patients with suspected (pregnancy test needed) or confirmed pregnancy, a decision has to be taken weighing carefully the risks versus potential benefits of the examination. Generally, it should be stated that there are no established emergency indications for PET/MRI. Concerning the radiation exposure caused by the PET-tracer application, please refer to the EANM PET/CT procedure guideline for tumor imaging as well as the International Commission on Radiological Protection (ICRP) [1, 30] or respective safety data that has been published for the particular tracer in use. Generally, and as long as the PET-tracer is labeled with ^18^F the exposure depends on the amount of radioactivity applied and ranges between 5 and 10mSv [31]. It should be noted that the overall exposure by a PET/MRI examination is significantly lower than by PET/CT since the radiation from the CT examination is omitted.

### Patient preparation and MRI safety

Preparation and instructions to patients depend on the tracer being injected and (at least for FDG imaging) are similar to PET/CT [1].

Additional specific instructions concerning MRI safety and patient preparation for MRI are defined by several international guidelines/papers [32–34]. All safety regulations and guidelines for MRI safety also apply to PET/MRI hybrid imaging. More specific, all established guidelines, patient questionnaires, and checklists for MRI safety need to be followed prior to an MRI or PET/MRI exam. This includes screening for potential MRI safety contraindications (e.g., claustrophobia, passive and active implants, metallic inclusions, pregnancy) [32–34]. Regarding MRI safety, it has to be noted that all current PET/MRI systems operate at 3-Tesla static magnetic field strength. This may have practical impact on MRI safety when scanning patients with MRI conditional cardiac pacemakers or other active implants is planned. Additional local rules regarding MRI and/or PET safety may apply.

Regarding safety, there is actually no overlap between the imaging modalities, which would save any preparation or safety step in PET or in MRI. However, it is crucial that all personnel involved in PET/MRI is familiar with both sides of PET and MRI preparations and safety instructions. It is also advisable to have a responsible safety person per imaging modality (in PET and MRI separately). If not possible, a “combined” safety officer can be established.

### Patient positioning and tracer and contrast administration schemes

The tracer administration scheme follows PET/CT recommendations. However, current PET/MRI systems incorporate highly efficient and sensitive PET detectors which allow for significant reduction of the activity of injected radiopharmaceutical, at least for ^18^F-based tracers [35–37]. This should be considered and incorporated in local protocols in accordance with the imaging time of the MRI.

Contrast agent administration is not influenced or altered by the concomitant PET acquisition. Thus, the standard MR-contrast choice (vendor) and injection protocol is in general not different from stand-alone MRI and depends on the institution’s preference. However, it has to be noted that the acquisition of all MR-based AC (MR-AC) has to be completed before commencing MRI contrast agent injection. Otherwise, tissue segmentation based on MRI images may lead to faulty results in MR-based AC methods [38].

Dependent on the tracer and investigational protocol involved, patients should arrive to the PET/MRI site between 60 to 90 min prior to the scheduled procedure start, allowing sufficient time for final anamnesis and to obtain informed consent of the patient or the care giver. Depending on the radiopharmaceutical and the investigational protocol, the patient gets the tracer injected either in advance to being positioned on the system or after it in the case of dynamic imaging studies. In the former case, imaging can start as soon as the accumulation and resting time has elapsed, the patient was asked to void, and the preparation of the system with all peripheral equipment is completed.

Then, the patient is positioned head first supine (at least mostly for whole body cases) on the patient bed, the radiofrequency (RF) head/neck coil and the flexible receiver coils and ear protectors are mounted and the patient is (again) instructed to keep still during imaging acquisition. The specific requirements in preparation for PET can generally be obtained from the respective clinical guidelines [16, 39]. Otherwise, patient positioning will depend on the clincal scenario, i.e., prone positioning of the patient in breast cancer cases.

In several cases, it is also possible that the MR-acquisition time might already start during the PET acquisition time so optimize the scanner room time, i.e., MR scanning can start at minute 40 with non contrast sequences during the PET-uptake time and the PET-acquisition is then started at minute 60.

### Attenuation correction and other correction methods

Different concepts and methods for scatter correction (SC) and AC in PET/MRI have been developed over the recent years. MR-based AC relies on the segmentation of MRI images into different tissue classes (e.g., background air, fat, muscle, lung tissue), based on their image-based grey scales. Following segmentation, the individual tissue compartments are then assigned a predefined LAC for the corresponding tissue [20]. Dedicated fast MRI sequences, mostly using the Dixon-technique providing fat and water images, are used to obtain images of tissue distribution and subsequent segmentation. This general method of tissue segmentation from MRI images is widely used in all currently available PET/MRI systems [40].

The method for AC of hardware components such as the patient table and RF coils for MRI signal reception is established by applying CT-based attenuation templates of the respective hardware components during PET data reconstruction [20, 41]. Such CT-based AC templates for the AC of most commercial RF coils are available on the current PET/MRI systems and are automatically considered during PET data reconstruction [20, 41].

Dedicated AC methods and MRI sequences have been developed to account for the increased attenuation of bone as additional tissue compartment as studies have suggested that not including bone could lead to errors in SUV determination of up to 25% [28]. In brain PET/MRI studies, the use of ultrashort echo time (UTE) or zero echo time (ZTE) sequences enables the consideration of skull bones in MR-based AC [42, 43]. For whole-body PET/MRI studies, the integration of bone-models allows AC of major bones [44–46]. More recently, deep learning methods have been developed to generate patient-specific synthetic CT images with bone information purely from MRI images [47]. These developments are currently very dynamic and will find their implementation into commercially available AC methods and applications in the near future.

To complete MR-based AC maps in whole-body PET/MRI examinations, methods for truncation correction are applied. These supplement the limited field-of-view in MRI that may lead to truncation of MRI signal along the patient arms in the MR-based AC maps. If not corrected, such truncations may cause faulty PET quantification following MR-based AC of PET data. Two general methods are used for truncation correction in PET/MRI. The maximum-likelihood reconstruction of attenuation and activity (MLAA) algorithm [48] estimates the outer contours of the patient body from non-AC PET images. Truncated areas in the MR-based AC map can thus be completed with information from PET images. This method for truncation correction is established on all currently available PET/MRI systems. However, it is limited to tracers like [^18^F]FDG that peripherally distribute throughout the body. A more recent and fully MR-based method for truncation correction is magnetic field homogenization using gradient enhancement (HUGE) [45, 49].

### Execution of examination (simultaneous procedures)

There are general types of investigational protocols for simultaneous PET/MRI. These are basically designed for simultaneous oncologic, neurologic, cardiologic, and inflammatory disease PET/MRI. Mostly in oncology but also in the other areas, there are special protocols adapted for pediatric PET/MRI. Moreover, there are protocol adaptions for specific body regions and disease entities like prostate cancer, breast cancer, or neuro-oncologic diseases. A comprehensive overview of oncologic indications that can profit from being diagnosed, staged, and monitored can be found in Umutlu and Hermann (eds.) [50] and there is, moreover, a multitude of clinical guidelines describing patient selection, preparation of imaging protocols, and analysis for/of oncologic PET investigations. In simultaneous PET/MRI, the PET part of the investigation will mostly be carried out as described in those aforementioned resources. Generally, the preparation starts with a thorough explanation of the imaging procedure to the patient and/or his/her caregivers. If claustrophobia related problems are to be expected, this might also include showing the system beforehand. In pediatric imaging [51] but also in some situations as described above as sedation or anesthesia might be necessary and the respective MRI-compatible equipment should be available. Depending on the tracer employed and the imaging protocol (static or dynamic PET imaging), the tracer is injected either before or immediately at the time the PET imaging protocol starts. Usually, the patient is positioned head-first supine on the system. In protocols other than just head/neck/brain, the RF spine coil, usually located in the patient bed, is always used. If the head region is of interest, the head should be placed and fixed inside the RF head coil. Moreover, region-specific, PET/MRI-compatible rigid or flexible receiver coils have to be fixed as close as possible to the body regions of interest. The imaging session usually starts with a localizer sequence with continuously moving table to plan the MRI sequences and the PET imaging. If tracer amounts according to diagnostic reference levels are used, the duration of PET imaging at one bed position or station usually is several minutes up to about 5 min. If dynamic imaging at only one bed-position is desired (i.e., in brain imaging), PET data acquisition in list mode would be the preferred methodology in order to be able to select time frames and durations later on while reconstruction. A basic oncologic set of MRI sequences should be for example comprised of a transverse half-fourier acquisition single-shot turbo spin-echo sequence (i.e., TE = 90 ms), a transverse diffusion-weighted imaging (i.e., b = 800 s/mm^2^), a coronal turbo inversion recovery magnitude (TIRM) sequence (i.e., TI = 220 ms) and possibly a three-dimensional magnetization prepared rapid acquisition gradient echo (T1) sequence. In the bed position of the thorax, a respiratory navigator is placed (manually or automatically, depending on the vendor) on the diaphragm and used for the MRI acquisition. Depending on the patient’s size, a scan of the whole body-trunk can thus be done within 20–30 min. Several modified whole-body protocols can be used as outlined in the individual indications below.

### Ending of examination, reconstruction, archive transfer, and releasing the patient

Immediately after the end of the simultaneous acquisition — i.e., while the patient is still on the table — visual quality checks of the acquired image data should be carried out to ensure and enable sufficient reporting and correct further data analysis. In particular, MRI images have to be checked for artifacts originating from patient motion, metal artifacts or RF artifacts. This also includes a thorough visual check of the generated attenuation map (µ-map) of LAC that is the basis for a quantitatively valid reconstruction of the PET data set. Meanwhile, there is a lot of different methods available that sufficiently solve the problem that structural MRI cannot directly deliver a µ-map as it does not yield an electron density signal [28, 41, 44, 52–58]. The aforementioned influences could generate artifacts that also could hamper the generation of a correct µ-map and, thus, impair the quantitative validity of the attenuation corrected reconstructed PET data set. If the acquired data are of good quality, the patient can be unloaded from the table. The patient should stay in the department waiting room as long as the reconstruction is not finished (only a few minutes). The PET reconstruction employs iterative reconstruction methods involving the attenuation and other corrections to the data using — if available — time-of-flight information in the PET event-protocols (sinograms). Moreover, recent software versions of the vendors enable truncation correction in outer body regions and use co-registered anatomical atlases, sometimes in combination with trained neuronal networks. That way, it improves tissue segmentation for MRI-based attenuation correction of PET data up to a level that in certain body regions performs as good and stable as the CT standards. This allows for a good representation of the actual body structure and property and, thus, its attenuation behavior against the 511 keV photons of the PET signal. The results (images) relevant to the clinical report should be transferred to a DICOM-compatible PACS system to be permanently stored. If the PET/MRI has been planned and carried out in the scenario of planning an external beam radiation treatment, ideally, the responsible physician immediately marks the target structures based on the molecular imaging results and sends those off to the treatment planning system along with the structural MR-imaging data set using the DICOM-RT structure set format. The patient can be released to the public and/or the referring ward after initial quality control of the acquired data are complete and satisfying.

### Motion correction

One of the potential advantages of PET/MRI over PET/CT is that simultaneous and independent PET and MRI data acquisition both require several minutes per bed position. What looks like a disadvantage at first sight can be turned into an advantage, namely providing motion correction of moving organs such as lungs and heart [59, 60]. Here, time-resolved MRI data can be used as a prior to correct for motion in PET data [59, 60]. Subsequent fusion of both data sets allows for display and reading of time-resolved lung and cardiac studies with the advantage that moving structures are depicted with less blurring and motion artifacts and with higher sharpness [60–62]. Best results in this context are obtained, when also the AC map is generated using motion correction. Then, the moving organs are considered in AC with each appropriate motion phase of the respective breathing and/or cardiac cycle.

In view of the relative complexity of PET/MRI attenuation correction and of the multitude and ever-growing number of AC methods available, it is recommended to always use the latest available software version and AC protocols on the respective PET/MRI system. Users are advised, furthermore, to strictly follow the most current recommendations of the manufacturers regarding AC methods and protocols. Finally, PET/MRI users are advised to carefully inspect non-AC PET data and the MR-based AC maps along with the AC PET data during image reading and reporting. Thereby, obvious motion artifacts [63] and artifacts around metallic implants are detected. If left unattended they may lead to faulty segmentation in the MR-based AC data, which then may result in local bias in PET data quantification or even visible artifacts in the PET data [64, 65]. Where available, TOF-PET detection shall be used to mitigate the quantitative impact of artifacts caused by metallic implants [66]. Further comments and insights on artifacts will be discussed below in the manuscript in the dedicated section.

### Protocol/image acquisition/workflow

Like in PET/CT, there are several workflow options especially on the MRI part of PET/MRI. First, it has to be decided if whole body PET/MRI (skull to upper thighs or even down to the distal lower limbs) or single station/partial body (between one and several PET positions) is needed to answer the clinical question. Single station (or few stations) imaging is possible even in oncologic indications (in contrast to cardiac and brain imaging where single station imaging is the default) since the injected tracer activities can be reduced and acquisition time for the PET can be adapted depending on the MRI protocol being used. Therefore, the current argument that once a tracer is injected (with its consecutive radiation exposure), the entire body should be imaged is, depending on the clinical question/area examined, partly not valid anymore.

Diagnostic MRI protocols should encompass all sequences and contrast weightings needed to answer the specific diagnostic question. In general terms, high quality MRI images should provide high soft tissue contrast, high spatial in-plane resolution, high spatial through-plane resolution (e.g., thin slices), homogeneous signal distribution and low image noise, and be free of any metal and motion artifacts. In integrated PET/MRI systems, MRI data is acquired simultaneously to PET data acquisition. For most efficient workflow, the data acquisition times for PET and MRI should match as closely as possible. This implies to reduce the number of MRI contrast weightings and sequences per bed position to the diagnostically necessary minimum.

The currently used PET/MRI protocols share several common technical aspects. MRI localizers are always required for the MRI acquisition planning. This initial planning process defines the axial range for the simultaneous PET/MRI examination. Current PET/MRI systems use single bed positions of about 25 cm, with a certain percentage of overlap between bed positions (depending on the vendor). Then, specific MRI sequences for attenuation correction have to be acquired, please refer to the respective section above. Finally, diagnostic MRI sequences for either whole-body or partial body imaging are the longest part of the protocol.

As indicated above, acquisition time can be adapted based on the MRI acquisition time and counterweighed vs. the injected activity. Since the MRI time is usually the dominant factor for the overall imaging time, it is expected that the injected tracer activity can be reduced significantly.

However, overall current literature suggests that the standard imaging time used in PET/MRI can be similar to PET/CT (2–4 min per bed position) with significant variations depending on the protocol [67].

The more frequently used protocol options are as follows:

- First, the general whole-body PET/MRI protocols, which should answer the most current and frequent medical questions. They are referred to as “standard PET/MRI” protocols.

These examinations can range from fully diagnostic PET/MRI protocols (e.g., with contrast media, longer scan times on specific organs), comparable to fully diagnostic contrast-enhanced PET/CT, to very short non-contrast protocols. Overall, within this category, protocol acquisitions have been divided in the literature into “ultrafast” or “basic” and “abbreviated” protocols which only use a fraction of the diagnostic MRI sequences which would normally be used in standalone MRI protocols [68–71].

Those protocols focus mainly on the information from PET component with only minimal basic MRI sequences simultaneously acquired [68] and thus comparable to a low-dose unenhanced PET/CT. The literature has reported such protocols being used for quick whole-body staging in anatomically “simple” diseases (i.e., lymphoma) or in patients with possibly low compliance or pain. Also, these protocols can be used for whole-body overview/metastases search integrated into more diagnostic/advanced protocols (see below). Basically, such acquisitions comprise of the Dixon-based attenuation sequences, possible additional higher resolution T1-sequences (Dixon or gradient echo sequences) and fast T2-sequences (with or without fat saturation).

**Fig. 1:**
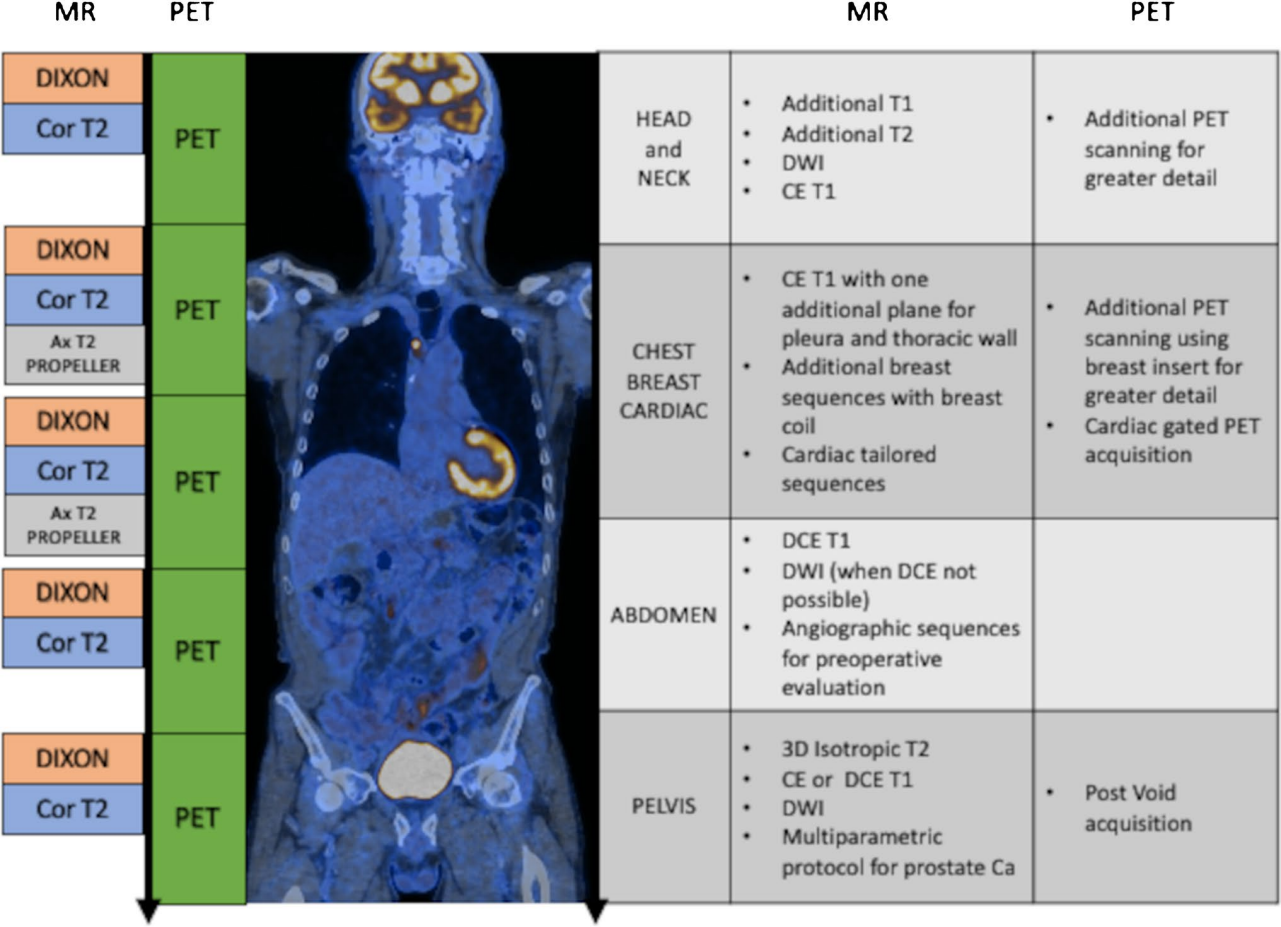
PET/MR workflow schematic. On the left of the PET/MR fusion image, the basic protocol similar in time and functionality to PET/CT is shown. To the right of the fusion image, examples of extra MR sequences and accompanying PET acquisitions that would exploit PET/MRs full potential are given. The latter, as explained in the text, would add time to the study making it longer than standard PET/CT

Those protocols (Fig. 1) are making maximal use of the complementary nature of the MRI and PET-derived information.

The next category has been called “fast” or also “basic” protocols in the literature [72]. Those still contain only whole-body sequences but with certain additions, i.e., fast T1 gradient-echo sequences post contrast, additional whole-body diffusion or additional fast T2-sequences in multiple planes. The extra sequences can be used to screen for small lesions otherwise possibly overlooked on other sequences, diffusion imaging being the most prominent example. Additional sequences in other planes (sagittal or coronal) contribute to localization of lesions/findings in anatomically challenging body compartments.

The next category is comprised of “fully diagnostic”, “advanced” or “dedicated” whole-body PET/MRI protocols. Those protocols would include again a “basic” whole-body overview, but specific additional fully diagnostic MRI sequences in the respective anatomical area (i.e., head and neck cancer protocols, chest protocols with gated sequences and/or UTE/ZTE, liver and pelvic protocols). It should be noted that even those fully dedicated MR-protocols can partly be abbreviated compared to their stand-alone MR-only counterparts [73]. Reasoning is that MR-only protocols are designed to provide the highest specificity and sensitivity for one imaging modality whereas hybrid imaging offers additional complementary information. As those fully diagnostic MR-protocols take somewhat longer based on the required MR-sequence acquisition time (even when shortened), they offer at the same time the possibility to acquire high-count, higher quality PET-imaging as well. These dedicated PET-images offer increased diagnostic accuracy especially in anatomical areas prone to breathing artifacts (chest, upper abdomen), where there is significantly increased physiological background activity (liver, lymphproliferative tissue of the head and neck) or where multiple areas of physiological uptake can make diagnosis challenging (pelvic exams with uptake in the bladder and bowel loops).

A further option is acquiring a specific, “local PET/MR” after a PET/CT in cases where specific questions are left unanswered. This could be, e.g., local extent of soft tissue tumors (where the MRI component has its clear advantages) or better PET image quality in previously questionably PET-positive lesions based on the potentially significantly longer PET acquisition time which can be used in such protocols.

A particular focus shall be given to dynamic PET/MRI protocols. In these situations — as stated before — patients are positioned with the body region of interest both axially and transaxially centered in the PET FOV. The goal is to follow the tracer distribution and metabolism over time in regions or organsof interest. There is a focus on dynamic brain PET but also for investigations of the heart or liver region, there are dynamic PET-imaging protocols available. Time resolution is achieved by subdividing the acquisition into predefined frames of time or acquiring in listmode to be able to decide on the time framing of the PET acquisition later on. In such dynamic PET acquisition protocols, the i.v. application of the tracer is performed parallel to the start of the PET-acquisition; although in practice, it is advisable to start the PET acquisition first and inject the tracer 1 min later to validate the positioning prior to tracer injection. Reconstruction can then be performed with 1 min delay. Depending on the intended data analysis, the application is performed as a bolus or continuous infusion using an infusion pump to ensure a stable infusion rate. If desired, it might be necessary to simultaneously take samples of arterial blood to enable full kinetic modeling of the tracer to characterize its pharmacokinetics. In neuro oncology, it is of importance to characterize the pharmacokinetic behavior of particular tracers as a parameter in staging the risk potential of tumors, such as for instance glioma [39, 74].

The last major option refers to “CT/MR-guided PET/MRI” protocols. Those are cases where a PET is warranted/indicated, but where there is already previous imaging for staging purposes available, and only very specific questions are left for PET/MRI. Thus, one can focus on the PET part for the whole-body examination (“low-dose PET/MRI”) and only add few specific MRI sequences on the region of interest, like the case of brain imaging in primary staging for lung cancer.

### PET image reconstruction

With PET being a quantitative imaging method with high sensitivity, PET data acquisition and reconstruction needs to fulfill numerous preconditions in order to provide accurate and quantitative results. PET data needs to be corrected regarding radiotracer decay over time, photon scatter, field-of-view truncation, and photon attenuation. Various methods for PET data correction have been established. We refer the interested reader to the section on attenuation correction of this paper and also refer to the detailed description on PET corrections and PET image reconstruction in the PET/CT guideline [1].

In the context of PET/MRI, specific topics have to be considered regarding PET image reconstruction. PET image reconstruction should be performed according to the manufacturers recommended standard reconstruction parameters (e.g., OSEM with appropriate number of iterations and pre-filtering). Furthermore, resolution modeling (point-spread-function (PSF) modeling) or other image processing and reconstruction methods may be applied depending on the scanner capabilities. Time-of-flight (TOF) PET information should be used during reconstruction, when available. It has to be noted, however, that all listed PET reconstruction parameters (e.g., OSEM, number of iterations, PSF modeling, TOF information) may have potential impact on PET quantification. This may have clinically relevant impact in repeated PET/MRI studies, where patients undergo repeated PET/MRI examinations for therapeutic monitoring or other indications. Similarly to PET/CT [1], in such PET/MRI studies, identical acquisition protocols and reconstruction parameters should be used.

As in PET/CT [1] and as mentioned above, it is good clinical practice to perform reconstructions with and without attenuation correction to be able to visually identify potential reconstruction artifacts caused by MR-based AC. Both attenuation-corrected (AC-PET) and non-attenuation-corrected (NAC-PET) images should be available for interpretation and lesions seen on the AC-PET images may need to be checked on the NAC-PET images, particularly when adjacent to metal implants or other artifact causing structures in the MR-based AC maps.

More technical details on the topics above can be found in a comprehensive review by Vandenberghe and Marsden [75] or Sattler [76].

### MRI image reconstruction

As mentioned in the “attenuation correction” section, MR-AC should be acquired and reconstructed according to the most current available version and according to the recommendations of the manufacturer. MR-AC µ-map should be carefully inspected during the PET data reading and reporting process.

Reconstruction of MRI images can be performed in any spatial orientation that is needed to match and overlay the PET data during PET/MRI hybrid image reading. It has to be noted, however, that for most 2D sequences, the orientation of MRI data acquisition (e.g., transaxial, coronal, sagittal, oblique) determines the direction of the highest spatial resolution. That is, a stack of transaxial 2D slices will provide high spatial in-plane (transaxial) resolution while the through-plane resolution in slice direction is reduced. Consequently, a sagittal or coronal reconstruction or reformatting of a transaxial 2D stack will show reduced spatial resolution that might hamper accurate anatomic assessment of findings in PET. Accordingly, the diagnostic MRI protocol for each bed position should include 2D MRI sequences with thin slices or 3D sequences with nearly isotropic spatial resolution.

## PET/MRI protocols based on clinical indications

### Neuro-oncology applications

In general, simultaneity is a convenient one stop-shopping approach, but not mandatory for neurological indications, as vendor provided co-registration software solutions between PET and MRI (if done in close temporal proximity) are usually sufficiently robust for clinical use in oncology cases. One exception to this might be the need for anesthesia, in which case sequential studies could entail either longer or repeated anesthesia, increasing patient risk [77, 78].

The most used PET tracers for brain tumors are described in Table 1.

**Table 1:** Most commonly used PET tracers for brain tumors

Tracer	Pathologies
2-deoxy-2-[^18^F]fluoro-D-glucose (FDG)	First choice: • CNS lymphoma Alternative use: • Glioma • MTS
O-(2-[^18^F]fluoroethyl)-L-tyrosine (FET) L-[methyl-^11^C]methionine (MET) 3,4-dihydroxy-6-[^18^F]fluoro-L-phenylalanine (FDOPA)	First choice: • Glioma
[^68^ Ga]DOTA-conjugated peptides	First choice: • Meningioma

Usually, a static amino-acid PET image acquisition of 10–20 min is sufficient for clinical use, while a 40–50 min dynamic acquisition for FET may provide additional diagnostic information (see imaging protocol below). Of course, if MRI acquisition is longer than 10–20 min, continued acquisition of PET information throughout the entire study can improve the quality of the PET images, depending on the properties of the used radiopharmaceutical. Further details on FDG and amino acid tracer patient preparation, image acquisition, reconstruction, semi-quantitative parameters, interpretation, reporting, and pitfalls are presented elsewhere [39]. A wide range of PET attenuation correction techniques in neuro PET/MRI has been suggested. The reported impairment of PET quantification by AC-artifacts in brain PET/MRI is, except for close proximity to bony structures or air cavities as low as known from PET/CT, on the order of up to 4%. Depending on the proximity to bony structures or air cavities, this may lead to systematic differences in the activity distribution and calculated semi-quantitative tumor metrics [79], which should carefully be considered during PET image interpretation [28, 80].

The MRI sequences required may depend on whether the patient has recently had extensive imaging, in which case an abbreviated protocol, possibly even only sequences for MRAC, might be acquired. Ellingson et al. [81] have proposed consensus guidelines for MRI of brain tumors for clinical trials, to assure reproducibility and inter-trial comparisons. They suggest 5 key sequences (3D T1 pre, Ax T2 FLAIR, Ax DWI, Ax T2 post-contrast, and 3D T1 post-contrast) as minimum standards [82]. To acquire all these sequences, an imaging duration of about 30 min is required. Examples of pediatric neurooncology PET/MRI protocols can be found in the literature [77, 78, 82, 83].

A number of advanced functional MRI techniques that may be useful in neurooncology in a multiparametric setting that are available for measurement of tumor perfusion by arterial spin labeling (ASL), tumor biochemistry by MRI spectroscopic imaging (MRSI), and tumor angiogenesis by relative cerebral blood volume (rCBV) using dynamic susceptibility contrast (DSC) or dynamic contrast enhancement (DCE) have been reviewed recently [84, 85]. Also, dedicated fMRI techniques can be performed for evaluation of feasibility of surgical resection in one setting together with PET-imaging.

An additive diagnostic advantage of combining advanced MRI techniques to standard PET and MRI has been suggested in smaller patient series [86], but has not been consistently documented. The challenges are the lack of standardization and availability of MRI techniques, both for acquisition and post-processing, and the limited tissue coverage because of susceptibility to patient movement and artifacts on postsurgical MRI [87, 88].

Other useful advanced MRI techniques for multiparametric use in neurooncology PET/MRI have recently been reviewed and published in a position paper by the European Cooperation in Science Technology (COST) Glioma MRI Imaging 2.0 (GliMR) initiative.

### Head and neck

For head and neck tumors, mainly FDG is indicated as radiotracer. Exceptions apply to neuroendocrine tumors, paraganglioma, and medullary thyroid carcinoma (DOTA-conjugated somatostatin receptor targeting peptides, DOPA) [89–91], extracranial meningiomas (DOTA-conjugated somatostatin receptor targeting peptides) [92], differentiated thyroid cancer (I124), and parathyroid neoplasia (choline-based radiotracers) [93]. However, the use of non-FDG radiotracers in these indications is partly off-label in the USA and in Europe.

PET/MRI can contain a dedicated MRI protocol tailored to the head and neck region. PET/MRI can be used for the staging of head and neck carcinomas greater than clinical stage T2 / N2a. Also, lower neck tumor location (e.g., in the hypopharynx) and the presence of lower neck level (III, IV) nodal metastases should prompt whole-body staging (which can be done with PET/MR), owing to the higher risk of distant metastases in these patients [94]. Furthermore, in a study conducted by Chan et al. [95], PET/MRI outperformed MRI and PET/CT in T and N staging while providing similar performance for M staging. Whole-body imaging should cover the area from the skull vertex to the upper thighs and is usually accomplished with an axial T1-weighted Dixon-type sequence, which takes approximately 3 min. With the lung being the most common site for distant metastatic disease, the acquisition of specific lung MRI sequences is recommended [96–98].

PET/MRI is useful for detecting residual disease in patients after non-surgical therapy, with optimal diagnostic reassurance at 12 weeks after therapy [99, 100]. Recurrent disease after different kinds of therapy can reliably detected with PET/MRI, as shown by Queiroz et al. [101].

PET/MRI for head and neck cancers can be safely performed without the use of gadolinium-based contrast agents. As shown by Kuhn et al. [102], this is still as accurate as contrast-enhanced PET/CT imaging, provided fat-suppressed T2-weighted pulse sequences are used in the head and neck region. However, for optimal results in the head and neck — and to obviate a separate MRI acquisition — PET/MRI should additionally contain at least a non-suppressed T1-weighted sequence before contrast and a fat-suppressed post-contrast T1-weighted sequence [94, 103]. As shown by Sekine et al. [103], such a protocol might be useful to determine the resectability of tumors. After contrast administration, the whole-body T1-weighted MRI scan might be repeated in patients with known or suspected distant metastatic disease or with other primary tumors.

During the MRI acquisition in the head and neck region, which takes approximately 20 min using the minimum protocol outlined above, one may keep the PET camera active to obtain a dedicated regional PET dataset with higher SNR. Functional MRI techniques aid in the characterization of tumors, lymph nodes, and suspected recurrences. However, their value in the setting of FDG-PET/MRI has not been studied in detail. Diffusion weighted imaging (DWI) as part of a PET/MRI protocol appears useful to detect unknown primaries and recurrent tumors after radiotherapy [100, 104, 105], but not for the staging of tumors [106]. The benefit of other functional MRI techniques as part of a head and neck PET/MRI protocol remains to be determined.

### Chest

When the local staging is already evaluated via the available chest CT, the PET/MRI protocol can be acquired with previously described “basic” or “fast” whole body protocols, including contrast media application [70, 71]. The PET acquisition time per bed position for those basic protocols have been described with usually 2–3 min which brings the PET/MRI acquisition time closely down to standard PET/CT imaging.

The brain MRI can be done as a standard brain MRI metastatic protocol, using the already applied contrast media for the whole-body acquisition. The PET-imaging time for the brain acquisition can be adapted to the MRI acquisition time. Alternatively, in cases of high-throughput PET/MRI prioritization, an abbreviated brain MRI protocol can be acquired to limit scanner time on the PET/MRI. In case there is a positive brain finding which explicitly needs further characterization, an extended brain MRI protocol can be acquired on a stand-alone MRI since the PET component usually does not add any advantage to the diagnostic accuracy in this scenario. With this approach, patients would have, in most instances, only one staging procedure instead of several different procedures. This is especially evident in health care systems with limited access to MRI and its consecutive waiting times.

Advanced PET/MRI protocols might be applied for local staging purposes in bronchial carcinoma and other chest malignancies (i.e., esophageal cancer). Several studies have shown, for example, that even in small pulmonary nodules, the lung parenchyma and mediastinal and pleural infiltration can be evaluated with advanced PET/MRI techniques (gating, UTE/ZTE, motion correction, radial Fourier plane acquisitions) [61, 107–112]. However, overall imaging time in the latter scenario will be significantly longer than that of standard PET/CT. Finally, data-driven motion correction techniques for PET reconstruction are also on the horizon [113–115].

Small initial studies comparing PET/MRI with PET/CT in local thoracic staging of malignant pleural mesothelioma (MPM) found a comparable diagnostic accuracy. Radiologists partly felt more confident staging with PET/MRI compared to PET/CT [116]. Overall, it was suggested that MPM can be staged using PET/MRI which is, however, always dependent on the local/jurisdictional circumstances [111].

### Breast cancer

PET/MRI has the advantage of being able to perform an accurate local staging in addition to regional and distant staging as discussed above. Dedicated breast MRI sequences and PET-compatible RF breast coils are available [117, 118]. Moreover, the physicians’ confidence in their diagnosis when interpreting co-registered PET and MRI images together can be increased [119–121]. Also, local staging can be achieved with significantly reduced radiation exposure [37]. It is currently of debate whether the dedicated breast acquisition with a dedicated breast coil in prone position should be performed in PET/MR. While this is the standard in MR-imaging, it might, however, not always be possible in PET/MR based on the more narrow patient tunnel.

There is also still debate if there is actually clinically added value of dedicated breast ^18^F-FDG PET/MRI for the evaluation of the primary breast cancer when compared to dedicated breast MRI. Newly developed FAP-imaging might offer new insights and improved diagnostic accuracy in breast cancer, also for the evaluation of the primary breast cancer [122–125]. There is already, however, consensus in the literature that ^18^F-FDG PET/MRI can provide added value for whole-body breast cancer staging and for treatment monitoring.

[^18^F]FDG PET/CT is more sensitive and MRI is more specific in predicting pathological complete response at the end of neoadjuvant chemotherapy, and therefore, both could be combined in order to improve such assessments.

Future directions include the use on other radiopharmaceuticals targeting estrogen and Her-2 receptor status, angiogenesis, or gastrin-releasing peptide receptor expression [126].

### Liver

Whole-body PET/MRI protocol can be combined with a liver specific PET/MRI protocol, which includes an additional, single-bed and (ideally) respiratory compensated PET acquisition of liver with simultaneous acquisition of liver specific MRI sequences. The MRI sequences have to be optimized for the specific indication, taking also into account the complexity and time constraints of simultaneous PET/MRI acquisition. Institutions may use gadoxetate (a paramagnetic contrast agent) for liver PET/MRI, depending on the indication and based on the higher accuracy of the hepatobiliary phase for oncologic liver imaging [127, 128].

There has been significant potential being demonstrated for [^18^F]FDG PET/MRI for imaging of hepatic metastases and to provide a potential one-stop-shop imaging solution for patients with known or suspected hepatic metastases [129, 130]. PET/MRI may improve reader confidence and the MRI component can improve characterization of focal hepatic lesions, especially PET negative metastases [131, 132].

However, there are only selected studies showing the advantage of PET/MRI in characterization of primary hepatic tumors. The main indication for [^18^F]FDG is the evaluation of tumor differentiation, i.e., in HCC and to predict hepatoma relapse after transplant. Thus, the main potential of PET/MRI for characterisation of primary hepatic tumors is likely to be realized with non-FDG radiotracers, i.e., choline, prostate specific membrane antigen (PSMA), FAPI, or CXC chemokine receptor type 4 (CXCR4)-based radiotracers.

### Pancreas

In pancreatic ductal adenocarcinoma (PDAC), MRI is equivalent to CT for the evaluation of local extension, vascular invasion, and nodal involvement. However, MR, compared to CT, has a higher sensitivity for the detection of small metastatic liver lesions (see above). Protocol requirements are relatively similar to PET/MRI of the liver. A whole-body PET/MRI overview can be followed by a single station/dual station PET-acquisition with simultaneous acquisition of pancreas (and liver) specific MRI sequences as per departmental guideline/as per published recommendations for MRI. The combination of PET and MRI, however, may be useful in assessing advanced imaging biomarkers. For example, regarding the evaluation of tumor aggressiveness, there is evidence of an inverse correlation between standardized uptake value (SUV) and apparent diffusion coefficient (ADC) in many malignant tumors as well as in pancreatic cancer [133]. In other studies, the metabolic tumor volume (MTV)/minimum ADC score ratio demonstrated the highest predictive ability for estimating the clinical TNM stage and was found to be an independent predictor of progression-free survival when done after treatment [134].

### Neuroendocrine tumors

MRI-only is an important modality for NET patients, as a large percentage of NET patients have liver dominant disease [135]. Gastro-entero-pancreatic NET primarily metastasize to the mesenteric lymph nodes and to the liver. The evaluation/characterization of liver metastases from NET should rely on a somatostatin receptor targeting radiopharmaceutical [136–138]. Indeed, SSTR-PET/MRI incorporating hepatobiliary phase imaging is a useful modality in patients with liver dominant NET. However, there is only very little data in NET and PET/MRI available [128, 139–143]. [^68^ Ga]Ga-DOTATOC PET/MRI can depict the anatomic correlates possibly better on the MRI component. However, lung lesions might be better seen with the CT component of the PET/CT.

There are currently no publications available concerning detection/characterisation of the primary tumor; however, for pancreatic tumors, this would not differ from what was described above. For NET’s of the small bowel, a specific MR-enterography protocol could be used in addition to the described basic whole-body PET/MRI.

Another potential role of SSTR-PET/MRI is in patients who have higher grade tumors. In higher grade NETs (increasing Ki-67 of 10% and higher), expression of the SSTR declines [144]. In this setting, diffusion weighted imaging can be helpful for lesion detection and characterization. Some facilities may perform both FDG and SSTR-PET-imaging for evaluation as the differential uptake can provide insight into tumor aggressiveness as well as response to SSTR-targeted therapies [145–148].

### Colon/rectal cancer

By integrating the quantitative parameters SUV and ADC from PET/MRI, an improved prediction and evaluation of therapy effect, compared with RECIST size measurements, may be achieved [149, 150].

In addition to the standard, above-described preparation, scopolamine butylbromide or glucagon can be additionally injected immediately before the start of investigation to minimize bowel motion and is used as a clinical standard in many institutions [151, 152]. FDG-PET/MRI in patients with colon tumors should contain a prolonged PET scan (12–15 min) in the area of interest in parallel with a detailed transverse DWI (at least 3 b-factors) and T1w and T2w sequences (with high spatial resolution) of the primary tumor.

Thereafter, a standardized protocol for whole-body MRI simultaneously with a whole-body FDG-PET acquisition (2–3 min. for each station) can be acquired for detection of distant colorectal metastases. DWI can be added to the standardized whole-body protocol, when the abdominal cavity is scanned, to improve detection of peritoneal carcinomatosis.

Contrast-enhanced MRI of the liver including T2w and DWI with 3b-factors using navigators for respiratory gating and T1w 3D fat saturated (breath hold) sequences before and after standard i.v. contrast or hepato-biliary specific contrast during arterial, portal phase and 4 and 10 min after contrast injection should be acquired.

Patients with rectal cancer are investigated with a standard clinical protocol according to the departmental guidelines but should include at least a T2-weighted sequence (in 3 planes) and DW (3 b-factors) images of the primary tumor in parallel with a prolonged PET scan in this bed position. The PET acquisition time can be adapted to the rectal MRI protocol. This again will be followed by the standard whole-body protocol [153, 154].

### Gynecological cancers

PET/MR has been shown to be beneficial for evaluation of cervical cancer, endometrial cancer, and ovarian lesions [155–160].

For gynecological cancers, the imaging protocol should be set up in consideration of the following aspects: (1) tumor entity (e.g., cancer of the uterine cervix or endometrial cancer); (2) desired coverage and clinical question — (a) primary local staging (pelvis only), (b) primary local and additional whole-body staging, and (c) whole-body restaging.

MRI protocols for local staging of primary tumors of the female pelvis (a) comprise a combination of T1- and T2-weighted sequences, dynamic imaging as well as potentially of diffusion-weighted imaging (exemplary protocol shown in Fig. 1). Optimal assessment of the different tumor entities (e.g., cervical cancer, endometrial cancer) requires protocol adaptations such as T2-weighted axial oblique plane imaging in case of cervical cancer to identify potential parametrial invasion [161, 162]. These protocol adaptations may be applied in accordance with current guidelines.

In case whole-body staging is desired in addition to local primary staging or for whole-body tumor relapse assessment, fast-whole body sequences as discussed above may be acquired for whole-body coverage [163, 164].

### Lymphoma

PET/CT-imaging is generally done for the following clinical indications: staging of FDG avid lymphoma (baseline), interim response assessment, and end of therapy response assessment [165, 166].

The Lugano classification, a consensus document developed following workshops at the International Conference on Malignant Lymphoma [167] has incorporated PET in the staging of all FDG-avid lymphomas.

The main purpose of initial staging is to accurately assess disease extent, at nodal and extra-nodal sites and to identify sites of bulky disease. Bulk has prognostic and therapeutic implications and therefore baseline maximal tumor diameter should be recorded.

PET-imaging based therapy response assessment criteria have a high negative predictive value of 95–100% for HL and 80–100% for aggressive NHL [168]. Residual lesions at interim assessment or after therapy are assessed with the Deauville score. Patients who do show residual disease at end of therapy, sites of positivity on PET may be used to guide biopsy to confirm residual disease prior to salvage therapy. Interim PET performed after 2 or 3 cycles of chemotherapy offers a window to the chemosensitivity of the tumor. A negative interim PET in Hodgkin’s lymphoma has been shown to indicate favorable response at end of therapy and in patients with advanced-stage HL, interim PET can be used to tailor management [169].

Routine PET/MRI protocol for staging of patients with lymphoma could include Dixon based attenuation correction sequences, a secondary plane with T2-sequence (with or without fat sat) and a 3D (fat suppressed) post contrast sequence. Overall acquisition time would be 25 min [72]. However, depending on the sequences used, this can be done also < 20 min. Unless contraindicated, there may be added value for contrast enhanced MR-imaging or DWI for better delineation of disease at extranodal sites, especially in bone marrow where MR-imaging has clear advantages over CT-imaging. Also, other extranodal lymphoma manifestations, i.e., brain or liver manifestations can be evaluated with improved accuracy on MR-imaging over CT-imaging.

Also, a special consideration for osseous involvement of hematological malignancies applies to PET/MR imaging in multiple myeloma [170]. The predominant bone marrow involvement in this disease makes PET/MR a well-suited combined imaging method for evaluation of this disease and PET- as well as MR-imaging are now already suggested in multiple myeloma classification [171].

For response assessment, protocols may be abbreviated even further, if a baseline scan exists. Dixon-based sequences for attenuation correction and the additional T2-sequence or 3D post contrast T1-imaging may suffice in identifying and measuring residual masses and assigning the appropriate Deauville score from the PET datasets. Since a decrease in size of mass along with negative PET has a better predictive of a favorable outcome as compared to either test alone, it is important to note and measure all residual masses along with metabolic response score [172].

### Prostate cancer

PET/MRI imaging is increasingly used in prostate cancer. The two main applications are detection and characterisation of primary disease within the prostate (mostly in high risk or the intermediate unfavorable risk group). Additional whole body imaging may be indicated for primary staging or in patients with biochemical recurrence [173–177]. PET/MRI acquisition is tailored according to the indication: primary staging, re-staging in cases of biochemical recurrence, and follow up after established diagnosis. For primary staging, a dedicated MRI of the prostate (see below) with adapted PET-acquisition time of the pelvis plus a fast whole-body acquisition should be performed. For cases with biochemical recurrence (post prostatectomy), fast whole-body sequences and similar pelvic sequence types can be used but have to be adapted to the pelvis instead of the prostate. For follow up of cases with established diagnosis, a fast whole-body overview might be sufficient.

[^18^F]Fluciclovine is widely used in the USA for prostate PET/CT and PET/MRI. However, the majority of imaging sites currently work with PSMA (either ^18^F or ^68^ Ga-labeled) and a recent publication with a head-to-head comparison suggested that PSMA-imaging might be the favorable imaging compound [178, 179]. Moreover, PSMA has recently been approved in the USA as well.

A problem with all radiopharmaceuticals for prostate imaging is that they are not prostate/prostate cancer specific and thus uptake in benign lesions such as benign prostatic hyperplasia (BPH) might represent a pitfall. However, with the addition of dedicated MR-sequences in PET/MRI specificity can be significantly increased up to 0.96 [180, 181]. Those dedicated protocols should include dedicated T2 sequences, dynamic contrast enhanced T1 imaging and diffusion weighted imaging (DWI).

Another pitfall is urinary excretion of most of the PSMA tracers (except for the 1007 compound, [^18^F]fluciclovine, [^64^Cu]Cu-PSMA, and [^18^F]methyl/ethyl choline) that may lead to scatter impaired image quality [182]. Thus, updated PET data reconstruction algorithms that can correct for this are available today and need to be used in the context of PSMA prostate PET/MRI [183, 184].

When using PET/MRI in the setting of biochemical recurrence, the local/pelvic imaging needs to be adapted accordingly and is different than prostate MRI for primary staging. DCE-MRI can aid in the detection of local recurrence, which is a common location for recurrence in patients with post-radical prostatectomy or radiation therapy [185]. Additionally, diffusion-weighted imaging can be helping in cases of local recurrence. It might, however, be limited by artifacts in patients post-brachytherapy. It has been shown that the complementary information from PET and MRI can increase confidence in interpretation by delineating suspicious anatomical findings that correlate with frequently subtle metabolic findings in patients with early biochemical recurrence [186].

### Carcinoma of unknown primary (CUP)

There is broad evidence of the utility of FDG PET/CT in patients with CUP [187, 188]. There are only two studies on PET/MRI in CUP [104, 189]. The studies found either comparable diagnostic accuracy between PET/MRI and PET/CT or higher detection rate for the actual primary tumor. It has to be noted that studies on CUP usually (as in this two cited studies) are studies about head and neck cancer. In those cases, as above described, a “fully diagnostic,” “advanced,” or “dedicated” PET/MR of the head and neck is recommended as described above. Given the time required by the MR for the diagnostic MR-sequences for the head and neck, the prolonged imaging time in PET allows how increased sensitivity and therefore possible detection of smaller primary head and neck tumors (i.e., in the tonsils).

However, other indications can occur as well, i.e., liver or lung metastases without an immediately found primary. Thus, the specific PET/MRI protocols in the respective anatomical areas as discussed in this guideline should then be used.

## Image interpretation, quantification, and reporting

### Artifacts in PET/MRI and their correction

The complexity of integrated PET/MRI hybrid imaging bears high potential for the occurrence of either PET, MRI, or PET/MRI-related artifacts [16, 190]. Beyond the mere visual affection of hybrid images, artifacts in PET/MRI may also have a significant effect on quantification of PET data. Since attenuation correction in PET/MRI is mostly based on MRI sequences, all artifacts in the MR-AC images will ultimately translate into inaccurate values in PET quantification following MR-AC [64, 191]. 

Typical artifacts in PET/MRI hybrid imaging are the following: motion artifacts and local misalignments between PET and MRI data due to patient and organ motion [192]; faulty tissue segmentation with assignment of wrong LAC in MR-based AC [64]; signal truncations along the patient’s arms where the patient anatomy often exceeds the spatial constraints of the MRI field-of-view [40, 193, 194]; and dental and metal implants that are found in a large and increasing group of patients [79, 195–197]. Apart from the safety aspects of metal implants that have to be clarified before any MRI and PET/MRI examination (see section on MRI safety above), all metal implants might cause signal voids or local distortions in diagnostic MRI images and in MRI-based AC that exceed the physical implant volume.

Recent studies now report about new developments to correct for artifacts. To reduce the quantitative impact of metal artifacts in MRI based AC on PET quantification, a method has been suggested to complete signal voids in the MR-AC caused by implants by deriving the shape and AC values of metal implants from PET emission data [198]. Truncation artifacts can be corrected by estimating the patient body contours from PET data using the MLAA algorithm [199]. A second method for correction of truncation artifacts is fully MR-imaging based and applies B0-HUGE to effectively increase the lateral field-of-view in MR-based AC [1, 45, 49]. In the context of metal artifact reduction in PET/MRI, it has been shown that TOF PET detection with fast PET detectors allows for a significant visual reduction of artifacts in the µ-map [66, 200], albeit PET quantification may still be biased. 

Regarding the management of artifacts in a current clinical PET/MRI setting, clinical users of PET/MRI are advised to always use the newest available generation of software and MR-AC methods. Time-of-flight detection shall be used were applicable to reduce the visual and quantitative influence of artifacts. Furthermore, image readers are advised to always carefully check the MR-based AC image data for artifacts during PET/MRI image reading. Tissue segmentation errors, Dixon technique related fat/water swaps, truncation artifacts, and large volume artifacts around metal implants are well detectable in the AC maps and indicate anatomic regions were the PET quantification may be hampered.

### PET quantification

PET quantitative reads may be required or are part of the PET/MRI study objectives. Although fully quantitative analysis using kinetic modeling approaches implying the ultimate need for dynamic PET imaging protocol (see above) may be considered most accurate, these studies are — apart from some neuro oncologic PET investigations — commonly not performed due to their complexity. Nevertheless, full quantitative studies may be required, e.g., to validate use of more simplified metrics derived from static imaging procedures as recently explained by Lammertsma et al. [201] and/or shown before by Cheebsumon et al. [202].

Typically, FDG uptake quantification is based on SUV or tumor/lesion background ratios (TBR). In these cases, tracer uptake in the tumor or lesion is normalized to injected activity over patient weight (or lean body mass [203]) in case of SUV or normalized to the uptake in a background region for TBR. The most applicable uptake metrics and how to obtain these have already been identified and recommended for oncology FDG PET/CT studies [1], but are equally applicable to PET/MRI. Similarly, recommendation for the analysis of FDG uptake in case of vascular diseases and for brain imaging are available and should be applied to PET/MRI studies as well [39]. In all cases when quantitative reads are desired, all necessary corrections to allow for quantitative reads should be included during the PET image acquisition and reconstruction process, such as normalization, dead time, decay, randoms, scatter, and attenuation correction. Dedicated sequences and procedures to derive MR-AC available on each PET/MRI system and as provided by the vendor should be applied.

### Reporting content and image interpretation

Detailed recommendations for reporting FDG findings have been published [1]. Niederkohr et al. published a reporting guidance listing the essential elements of a concise and complete oncologic [^18^F]FDG PET/CT report [204]. Recommendations are also provided in the European Guideline for FDG PET/CT oncology imaging. These recommendations refer to PET/CT but are equally applicable to PET/MRI regarding the reporting of patient history, details of the FDG imaging procedure, and any clinical findings and conclusions derived from the FDG PET/MRI study. In case of FDG brain imaging studies, recommendations have been published before and are similarly applicable to PET/MRI [39, 205].

In case of image interpretation of PET/MRI studies, particular attention is needed to potential MR-AC artifacts and pitfalls, as explained before. For both PET/CT and PET/MRI, attenuation correction artifacts may occur, although different in cause and nature. It is therefore recommended to not only inspect both the attenuation and non-attenuation corrected PET images but also to inspect the generated MR-based attenuation coefficient image (µ-image or µ-map) for any unexpected artifacts [1]. Due to differences in MR-AC versus CT-AC, differences in apparent tracer uptake distribution may occur. FDG uptake in and near bone may appear lower in PET/MRI than this seen in PET/CT. Furthermore, due to the assignment of a uniform attenuation coefficient to lung tissue, uptake in the healthy lung tissue may have a slightly different appearance. Several techniques became meanwhile available to correct for different MR-AC aspects. Irrespective which is used and despite potential differences in the quality and accuracy of MR-AC versus CT attenuation correction, clinical PET/MRI images show a very comparable physiological FDG distribution as those seen in PET/CT [1, 3].

### RADS/other reporting classifications

While there is no standard template to report hybrid imaging (PET/MRI or PET/CT for that matter), there is a wealth of (structured) reporting systems available in CT, MRI, and ultrasound. There are several RADS (Reporting and Data System) available (i.e., for HCC, prostate cancer, thyroid cancer, and many more). Although it has been shown that there is no influence on reporting quality or sensitivity of detection of the disease itself, RADS harmonizes the imaging/reporting output. The major values of such reporting and data systems are that it provides consistency in terminology, which makes the reporting more reliable and better understandable for the referring physicians. 

Since there are several reporting systems available for PET as well as for MR, either stratified by disease or even by therapy, it is not possible to give a concise recommendation which reporting system to use. However, it is justified that standardized reporting is used for both components (if available) in PET/MRI within the institutions’ preferences. This (institutional) standard shall comprise a joint final assessment and conclusion of each report including the approval of both the MRI- and PET-specialized MD.

## Data Availability

Data sharing is not applicable to this article as no datasets were generated or analysed during the current study.

## Declarations

### Liability statement

This guideline summarizes the views of the EANM Oncology-Theranostic Committee, SNMMI and ISMRM. It reflects recommendations for which the EANM, SNMMI and ISMRM cannot be held responsible. The recommendations should be taken into context of good practice of nuclear medicine and do not substitute for national and international legal or regulatory provisions.
